# Impact of an *Alu* insertion on the cellular localisation of tissue factor protein

**DOI:** 10.1038/s41598-025-19280-4

**Published:** 2025-10-10

**Authors:** Courtney Chatterton-Bartley, Jun Cao, Joseph Boyle, Dorian Haskard

**Affiliations:** National Heart and Lung Institute, Imperial College London, Hammersmith Campus, Hammersmith Campus, London, W12 0NN UK

**Keywords:** Cell biology, Evolution, Genetics, Molecular medicine

## Abstract

Tissue Factor (Hugo Gene Nomenclature Consortium HGNC gene Factor III (F3)) plays vital roles in many cellular processes including haemostasis, thrombosis, inflammation and angiogenesis. Approximately 30–40 million years ago, an inverted *Alu* element inserted into the *F3* 3′-UTR, and we hypothesised that this altered post-translational intra-cellular trafficking of *F3* protein. Confocal microscopy of MDA-MB-231 human breast cancer cells using fluoroprobe constructs regulated by the *F3* 3′-UTR with or without the *Alu* insertion demonstrated that the presence of the *Alu* element promoted protein localisation to the Golgi, and reduced cell membrane localisation. Furthermore, stimulation of cells with a Protease-Activated Receptor-2 activating peptide led to rapid mobilisation of the fluorophore from the Golgi to the cell surface. These findings suggest that the *Alu* insertion facilitates Golgi retention of *F3* and mobilisation to the cell surface upon demand. This mechanism may provide an evolutionary advantage by fine-tuning the cellular haemostatic response in primates.

**Supplementary Information:**

The online version contains supplementary material available at 10.1038/s41598-025-19280-4.

## Introduction

Tissue Factor (Hugo Gene Nomenclature Consortium HGNC gene Factor III (*F3)*) plays vital roles in many cellular processes including haemostasis, thrombosis, inflammation and angiogenesis^1^. It has a varied means of expression across different cell types. In vascular adventitial fibroblasts and epithelial cells, it is expressed constitutively and may act as a haemostatic envelope triggering coagulation in the event of tissue injury^2^.

The intracellular trafficking of *F3* allows rapid mobilisation to the cell surface when appropriate. Whilst much is known about the expression pattern of *F3* on the cell surface of different cell types, the subcellular localisation and dynamics have been less studied. In both growth factor stimulated smooth muscle cells and Baby Hamster Kidney cells transfected with exogenous *F3*, the majority of *F3* protein localises to intracellular pools in the perinuclear cytoplasm^3,4^. Mandal et al. showed that a large fraction of the intracellular pool of *F3* in human fibroblasts is localised to the Golgi^5^. Furthermore, they found that activation of protease-activated receptor-2 (PAR-2), a G-protein coupled receptor involved in various cellular responses, mobilises intracellular *F3* from the Golgi and increases its expression on the cell surface^6^.

**Fig. 1:**
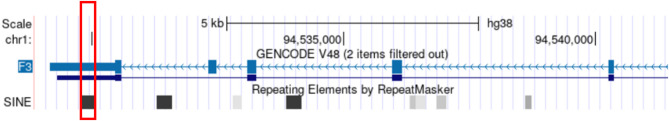
UCSC Genome Browser visualisation of the *F3* gene highlighting the location of the *Alu* element. UCSC Genome Browser snapshot of the *F3* gene on the human genome assembly (GENCODE v48, *hg38*). The GENCODE Genes track (top) illustrates the exon–intron structure of the gene and 3′-UTR, and the RepeatMasker track (bottom) identifies Short Interspersed nuclear elements (SINE), including the *Alu* element (highlighted by the red box).

It has been briefly reported that the *F3* 3′-UTR contains an inverted *Alu* element^7^ (Fig. 1). *Alu* elements are primate specific, short interspersed mobile DNA sequences^8^. They first appeared during primate evolution and are now recognised as potential modulators of gene regulation^9^. They can influence transcriptional and post-transcriptional processes through several mechanisms, including serving as alternative promoters, enhancers, and transcription factor binding sites, or altering DNA methylation patterns^9–13^. Differential expression of genes with *Alu* element insertions may play a role in embryo development^14^. Furthermore, *Alu* element insertion can lead to protein truncation and thereby alter function^15^. The functional importance and possible organismal advantage of the *Alu* element in *F3* 3′-UTR has not previously been addressed.

This study tested whether the inverted *Alu* element affects the subcellular localisation of human *F3*.

## Materials and methods

### Cell lines

The MDA-MB-231 (a human breast cancer cell line that expresses *F3* constitutively^16^ cell line were purchased from the ECACC.

### Confocal microscopy constructs

Fluorescent *F3* constructs (FC1, FC2, FC3, and FC4) were subcloned into the pcDNA™3.1/Zeo(+) vector by GeneArt. GFP-EEA1 wt was a gift from Silvia Corvera (Addgene plasmid # 42307; http://n2t.net/addgene:42307; RRID: Addgene_42307)^17^; mEmerald-Nucleus-7 was a gift from Michael Davidson (Addgene plasmid # 54206; http://n2t.net/addgene:54206; RRID: Addgene_54206, unpublished); PA-GFP Golgi was a gift from Michael Davidson (Addgene plasmid # 57164; http://n2t.net/addgene:57164; RRID: Addgene_57164, unpublished); GFP- SEC61B was a gift from Christine Mayr (Addgene plasmid # 121159; http://n2t.net/addgene:121159; RRID: Addgene 121159)^18^.

### Transfections

On the day of transfection, MDA-MB-231 cells were washed with PBS. Cells were then incubated in serum-free media (DMEM media with 1% L-glutamine; 500 µl) at 37 °C and 5% CO_2_ for 30 min. Opti-MEM™ (50 µl) was added to two wells in a 96-well plate per transfection reaction. 2 µl of Lipofectamine™ 2000 was added to one well of Opti-MEM™, and the 1 µg of DNA added to the other. The DNA-Opti-MEM™ was then added to the Lipofectamine™-Opti-MEM™ dropwise and left to incubate at room temperature for 10 min to form the DNA-Lipofectamine™ complex. The complex (100 µl) was then carefully added to the cells, after which the plate gently shaken to cover the cells and incubated at 37 °C and 5% CO_2_. After 5 h, 500 µl full DMEM media with 1% L-glutamine and 10% FBS was added to the cells and further incubated at 37 °C and 5% CO_2_, in a humidified tissue culture incubator.

### Confocal microscopy

MDA-MB-231 cells were seeded at a concentration of 5 × 10^4^ in 4-well glass bottomed slides, in base media, 24 h prior to transfection. Cells were washed with PBS and incubated at 37 °C and 5% CO_2_ in serum free media for 30 min. 0.5 µg of DNA was transfected into cells using Opti-MEM™ and Lipofectamine™ 2000. The cells were left at 37 °C and 5% CO_2_ for 5 h, when full base DMEM media was added.

CellMask™ Orange was used as a cell membrane marker. It was diluted to 2x working solution in pre-warmed base media and added to cells 30 min prior to imaging. Cells were incubated at 37 °C and 5% CO_2_.

The PAR-2 agonist peptide SLIGKV-NH₂ (R&D Systems) was reconstituted according to the manufacturer’s instructions and used at a final concentration of 100 µM. Cells were treated for the time period specified in the Figure legends. 24 h following transfection, media was changed for fresh base media. 0.5 µl of working solution Hoechst 33342 nuclear stain was added to each well, and the cells covered in foil to protect from the light and left for 10 min. Media was then carefully removed and replaced with FluoroBrite™ DMEM media ready for imaging.

Cells were imaged using a Zeiss LSM-780 inverted confocal microscope. The objective lens used is specified in the Figure legend. For live cell imaging, the chamber was heated to 37 °C with 5% CO_2_ conditions.

ImageJ software was used to visualise and analyse the images. An EzColocalization ImageJ plugin developed by Stauffer W., et al. was used to conduct all the immunofluorescence colocalization analysis^19^.

## Results

### Effects of the *Alu* insertion on *F3* post-translational protein distribution

**Fig. 2:**
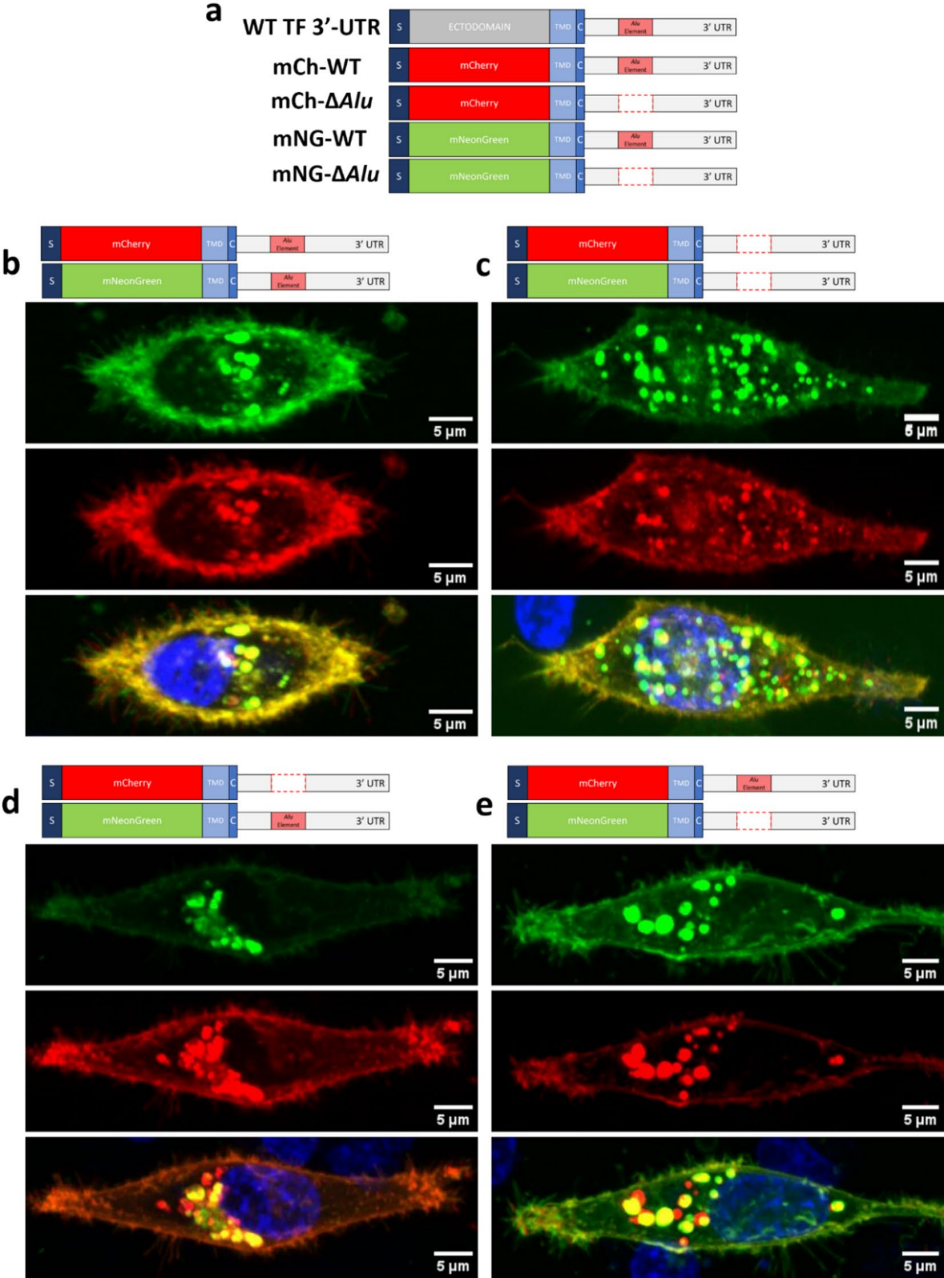
Co-transfection of WT and Δ *Alu* fluorescent constructs. (**a**) The top row shows the WT human *F3* 3′-UTR construct. For each of the fluorescent constructs the ectodomain has been replaced by either mCherry or mNeonGreen. The *Alu* element is highlighted by a red box in the 3′-UTR, and red dotted line indicates a deletion of the *Alu* element; (**b–e**) Fluorescent constructs were co-transfected into MDA-MB-231 cells. Images were acquired as a Z-stack using Plan-Apochromat 63x/1.40 Oil DIC M27 lens on Zeiss LSM-780 inverted confocal microscope. Representative of multiple images, shown as a Z-project. Scale bars, 5 μm. Images analysed on Fiji software (ImageJ). (**b**) mCh-WT and mNG-WT; (**c**) mCh-Δ*Alu* and mNG-Δ*Alu*; (**d**) mNG-WT and mCh-Δ*Alu*; and (**e**) mCh-WT and mNG-Δ*Alu*. Fluorophores encoded by the Δ*Alu* 3′-UTR *F3* constructs localise to the cell surface more readily than those encoded by the WT 3’-UTR *F3* constructs.

Fluorescent reporter constructs were designed with or without the *Alu* element in the human *F3* 3′-UTR (Fig. 2a) and expressed in cells to track protein localisation. Both constructs colocalise equally intracellularly and extracellularly if they have the same 3′-UTR (Fig. 2b and c), confirming neither fluorophore influences localisation. However, when constructs with different 3′-UTRs are dual-transfected (either mCh-WT/mNG-∆*Alu* or mNG-WT/mCh-∆*Alu*), the protein generated via the ∆*Alu* 3′-UTR showed more trafficking to the cell surface than that generated from the construct with the WT 3′-UTR (Fig. 2d and e). The overall distribution of mCh-WT *F3* 3′-UTR showed a similar distribution to endogenous *F3* protein (Figure S1).

**Fig. 3:**
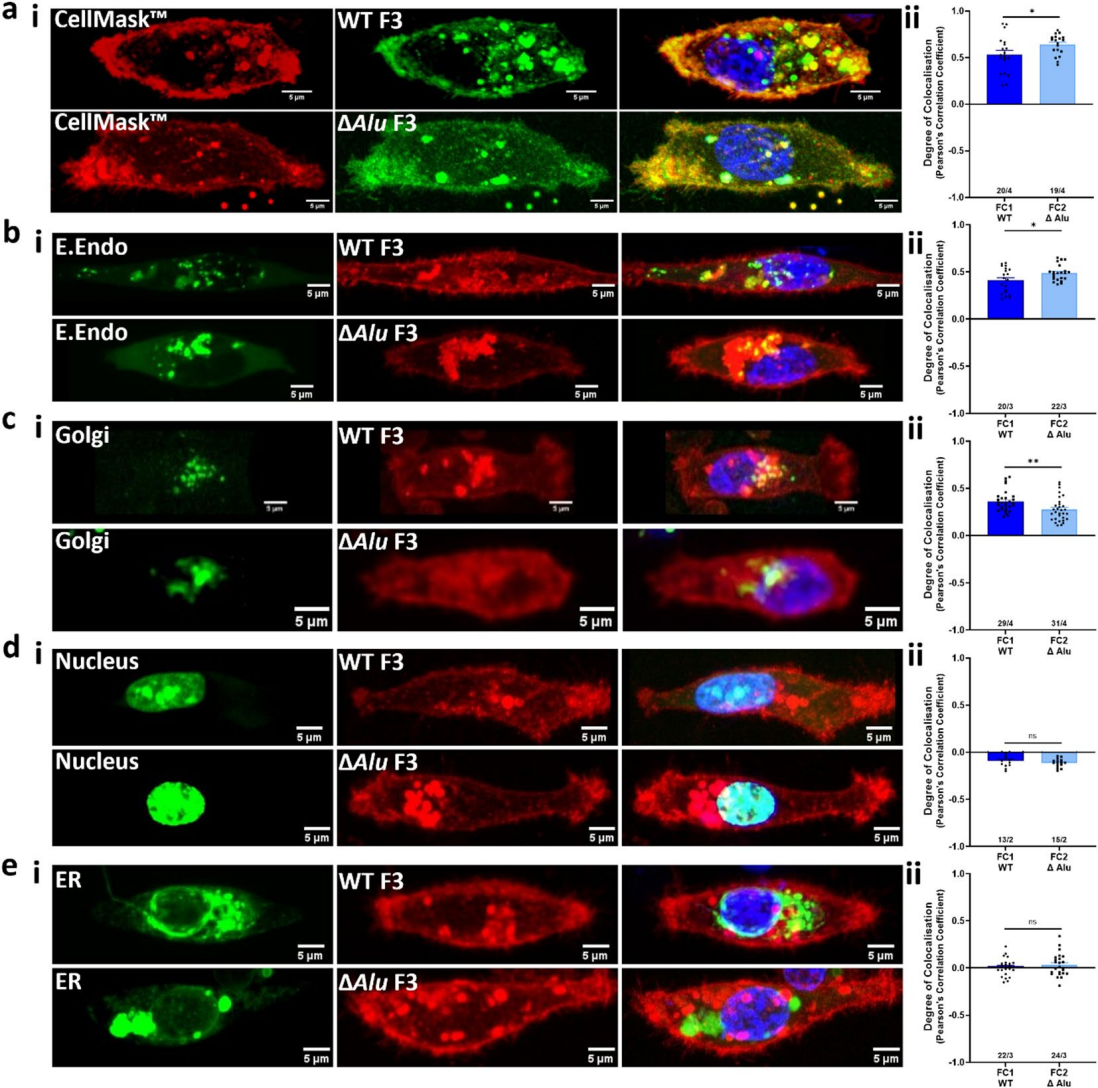
The presence of the *Alu* element in the *F3* 3′-UTR affects its localisation within MDA-MB-231 cells. MDA-MB-231 cells were co-transfected with markers to identify specific organelles along with a fluorophore under the influence of the *F3* 3′-UTR with either the *Alu* element present (WT) or not (∆*Alu*). (**a**) cell membranes, (**b**) early endosomes, (**c**) Golgi, (**d**) nucleus, (**e**) endoplasmic reticulum. (i) Left columns represent organelle localisation; middle column, WT or Δ*Alu F3*; and the right column, the merged image (colocalisation) of *F3*, the organelle marker, and Hoechst 33342 to identify the nucleus (blue). Images were acquired as a Z-stack using Plan-Apochromat 63x/1.40 Oil DIC M27 lens on Zeiss LSM-780 inverted confocal microscope. Representative of multiple images. Scale bars, 5 μm. (ii) Pearson’s correlation coefficient of the colocalisation of the organelle marker with the fluorophore protein under the influence of either the WT or Δ*Alu* 3′-UTR are shown to the right. The number of cells analysed/the number of independent experiments is indicated on the x axis. Data are expressed as mean ± SEM and analysed using one-way ANOVA. *p < 0.05 **p < 0.01.

We next looked in more detail at differential localisation using a panel of membrane and organelle markers and Pearson correlation analysis. ∆*Alu* constructs showed significantly greater colocalisation with the plasma membrane (Fig. 3a, Figure S2) and early endosomes (Fig. 3b, Figure S3) compared to WT 3′UTR constructs. Conversely, colocalisation of mCh-∆*Alu* with a Golgi marker was significantly less than the colocalisation of mCh-WT (Fig. 3c, Figure S4). There was no correlation of either mCh-WT or mCh-∆*Alu* with markers of the nucleus (Fig. 3d, Figure S5) or endoplasmic reticulum (Fig. 3e, Figure S6). Although to the naked eye the differences between constructs are subtle, significance is shown by the image analysis. Overall, this indicates that fluorescent proteins generated from constructs without the 3’-UTR *Alu* element (i.e. mCh-∆*Alu* and mNG-∆*Alu*) are more readily trafficked to the cell surface than those generated from constructs containing the 3’-UTR *Alu* element.

## Effects of PAR-2 stimulation

**Fig. 4:**
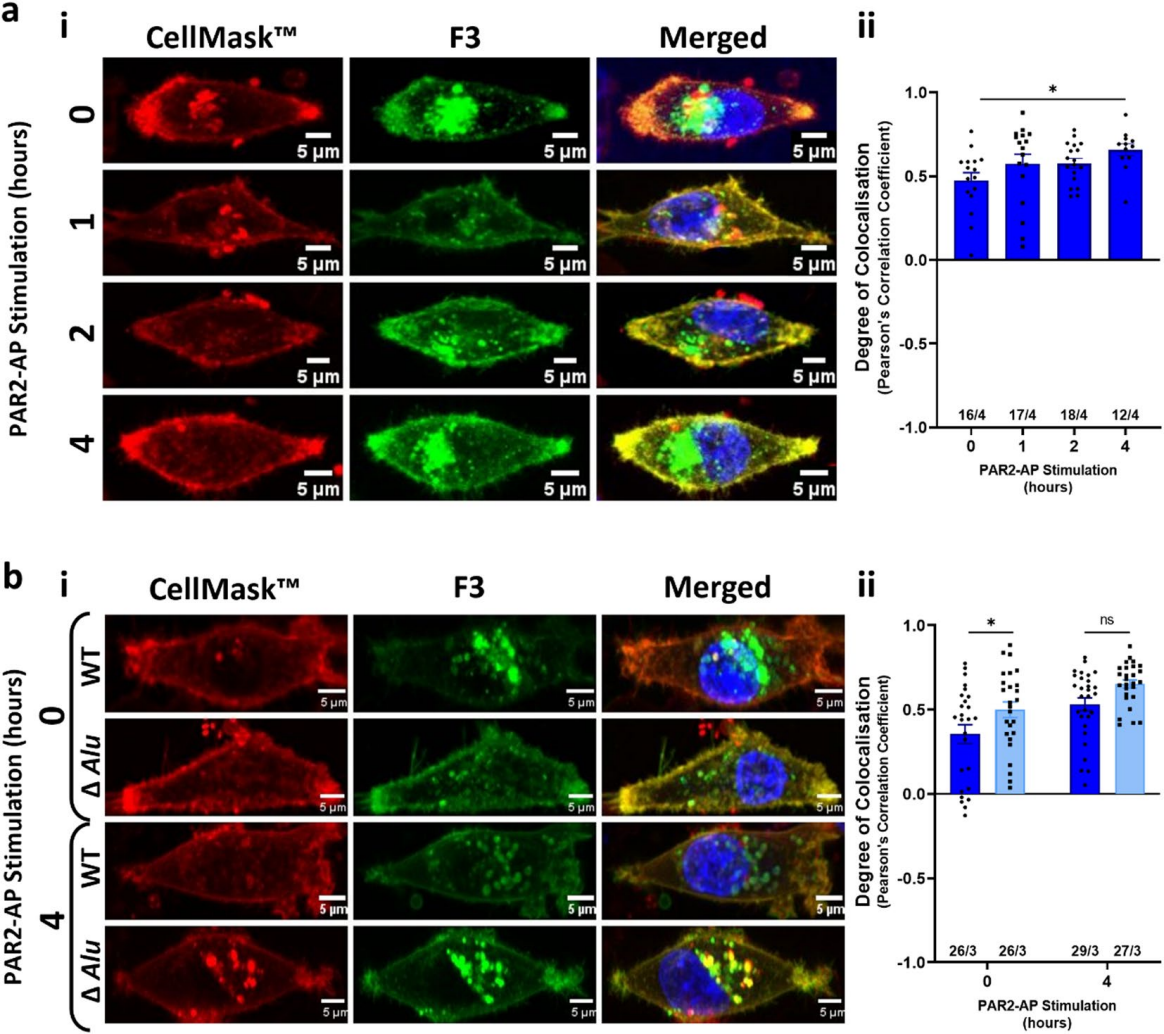
PAR2-AP increases *F3* protein to the cell membrane but has no effect when there is no *Alu* element present in the *F3* 3′-UTR. (**a**) Fluorescence confocal microscopy of live MDA-MB-231 cells after transfection of mNG-WT (green, left panels), following stimulation with PAR2-AP (0, 1, 2, or 4 h) and stained with CellMask™ Orange visualising plasma membrane (red), and Hoechst 33,342 (blue). (i) The left panel shows plasma membrane, the middle column shows the fluorescent *F3* constructs, and the right panel shows a merged image. Colocalisation of *F3* with plasma membrane appears yellow. PAR2-AP increases *F3* expression in the cell membrane. Images were acquired as a Z-stack using Plan-Apochromat 63x/1.40 Oil DIC M27 lens on Zeiss LSM-780 inverted confocal microscope. Representative of multiple images. Scale bars, 5 μm. (ii) Pearson’s correlation coefficient of the colocalisation of the plasma membrane with mNeonGreen protein under the influence of *F3*′*s* WT 3′-UTR are shown. The number of cells analysed/the number of independent experiments is indicated on the x axis. Data are expressed as mean ± SEM and analysed using one-way ANOVA. *p < 0.05; (**b**) Similar confocal microscopy after transfection of mNG-WT (green) or mNG-Δ*Alu* (green), following stimulation with PAR2-AP for 4 h and stained with CellMask™ Orange visualising the plasma membrane (red), and DAPI (blue). (i) The left panel shows plasma membrane, the middle column shows the fluorescent *F3* constructs, and the right panel shows a merged image. Images were acquired as a Z-stack using Plan-Apochromat 63x/1.40 Oil DIC M27 lens on Zeiss LSM-780 inverted confocal microscope. Representative of multiple images. Scale bars, 5 μm. (ii) Pearson’s correlation coefficient of the colocalisation of the plasma membrane with mNeonGreen protein under the influence of *F3*′*s* WT and Δ*Alu* 3′-UTR are shown. The number of cells analysed/the number of independent experiments is indicated on the x axis. Data are expressed as mean ± SEM and analysed using one-way ANOVA. *p < 0.05.

PAR2-AP (SLIGKV-NH2) is known to mobilise *F3* from the Golgi to the cell surface in fibroblasts^6^. Stimulation of MDA-MB-231 cells with PAR2-AP significantly increased the colocalisation of the mNG-WT with the cell surface (Fig. 4a). As the loss of the *Alu* insertion reduced fluorophore colocalisation with the Golgi, we asked whether the *Alu* element affected the response to the PAR2-AP in our experimental system. Whilst there was a significant increase of mNG-WT with CellMask™ following PAR2-AP stimulation (*p* = 0.0381), a corresponding increase was not seen for mNG-∆*Alu* (Fig. 4b).

## Discussion

We have shown that removal of the inverted *Alu* element from the *F3* 3′-UTR affects the subcellular localisation of *F3* protein. Using fluorophore reporter constructs, we found that in the absence of the *Alu* element there was increased plasma membrane and early endosomal localisation of the reporter and reduced presence in the Golgi. Human *F3* is known to localise to the Golgi, and to mobilise to the plasma membrane following cell activation by PAR-2 receptor ligation^5^. Our findings suggest that the *Alu* element may influence intracellular trafficking, potentially promoting Golgi retention of *F3*.

PAR-2 is a G-protein-coupled receptor involved in various physiological processes such as coagulation, inflammation, and tissue repair^20^. It is known to trigger *F3* mobilisation from the Golgi to the cell surface upon activation by proteases potentially present during tissue injury^5^. Consistent with this, we observed increased surface expression of the fluorophore upon PAR-2 activation, but only if the *Alu* element is present in the *F3* 3′-UTR. This supports the hypothesis that the *Alu* insertion enables enhanced Golgi storage of *F3*, allowing for rapid mobilisation in response to cellular cues. Such rapid mobilisation of an intracellular pool may be advantageous during acute injury or inflammation, enabling swift haemostatic responses.

One possibility is that the *Alu* may affect mRNA localisation and subsequent translation, a process that mostly takes place in the endoplasmic reticulum, but which can also take place in the Golgi^21^. Alternatively, there is the possibility that the *Alu* insertion in 3′-UTR may affect post-translational protein trafficking by affecting the assembly of proteins on *F3* during or after its translation. A precedent for such a mechanism comes from the work of Berkovits et al., which showed that alternative poly-adenylation of *CD47* mRNA alters the assembly of proteins on CD47 during or after its translation. This affects CD47 protein localisation to the plasma membrane without affecting mRNA abundance, isoform levels, or protein translation efficiency^22^. A further consideration is that the *Alu* element in the *F3* 3′UTR might act as a binding site for a non-coding RNA(s) that affects mRNA and/or protein localisation^23^.

In conclusion, our findings reveal that the *Alu* element in the *F3* 3′-UTR significantly influences the subcellular trafficking of *F3* protein, likely enhancing its readiness for mobilisation during haemostatic or inflammatory challenges. This suggests that the primate-specific evolutionary insertion has functional consequences that may provide a selective advantage. The presence of the inverted *Alu* element in the *F3* mRNA 3′-UTR could possibly enhance the dual function of *F3* in coagulation and infection control. More generally, altered intracellular trafficking should be added to the possible effects of *Alu* insertions on gene expression.

## Supplementary Information

Below is the link to the electronic supplementary material.

Supplementary Material 1

## Data Availability

The datasets used and/or analysed during the current study are available from the corresponding author on reasonable request.
