# Supplementary material for: Impact of an Alu insertion on the cellular localisation of tissue factor protein
Source: Sci Rep. 2025 Oct 10;15:35488. doi: 10.1038/s41598-025-19280-4 (PMC12514209; doi:10.1038/s41598-025-19280-4)
Supplement: Supplementary file 1 — Supplementary Material 1 [file 41598_2025_19280_MOESM1_ESM.pdf]

Supplementary Material and Methods

1. **Endogenous F3 Confocal**

24 hours following transfection, cells were carefully washed twice with room temperature PBS. 4% formaldehyde solution was carefully overlayed onto cells and left at room temperature for 15 minutes. Cells were washed with PBS and then blocked with 200 µl 1% BSA/PBS. APC conjugated F3 antibody was diluted in 1:1000 in PBS. 100 µl of primary antibody solution was laid over cells and left to incubate for 1 hour, covered in foil to protect from the light. Cells were washed 3 times in PBS. DAPI 1 mg/ml was diluted 1:1000 in PBS. 0.5 µl was added to cells and incubated for 5 minutes. Finally, the cells were washed with three times in PBS before leaving the cells in 500 µl PBS for imaging. ImageJ software was used to visualise and analyse the images. An EzColocalization ImageJ plugin developed by Stauffer W., et al was used to conduct all the immunofluorescence colocalization analysis[17].

Supplementary Figures

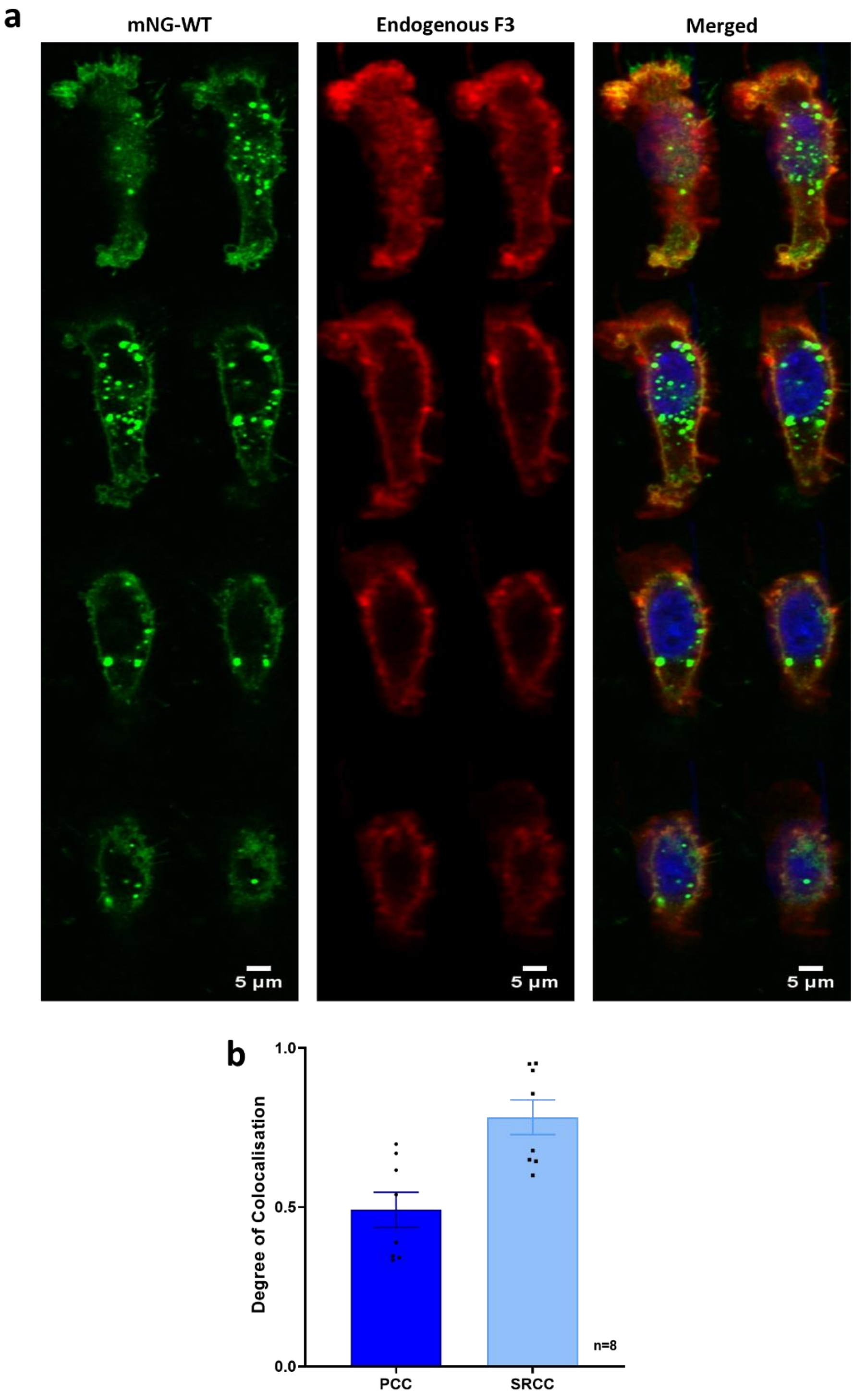

Figure S1. mCh-WT localises with endogenous F3 protein.

a. MDA-MB-231 human breast cancer cells were transfected with mCh-WT (red) and then antibody-stained for endogenous F3 (green). Colocalization appears as orange in the merged image (right). Images were acquired using Plan-Apochromat 63x/1.40 Oil DIC M27 lens on Zeiss LSM-780 inverted confocal microscope. Shown are 8 representative slices of the Z-stack. Representative of multiple images. Scale bars, 5 µm.
b. Pearsons Correlation Coefficient (PCC) and Spearman's Rank Correlation Coefficient (SRCC) for the colocalisation of exogenous and endogenous F3 protein are shown.

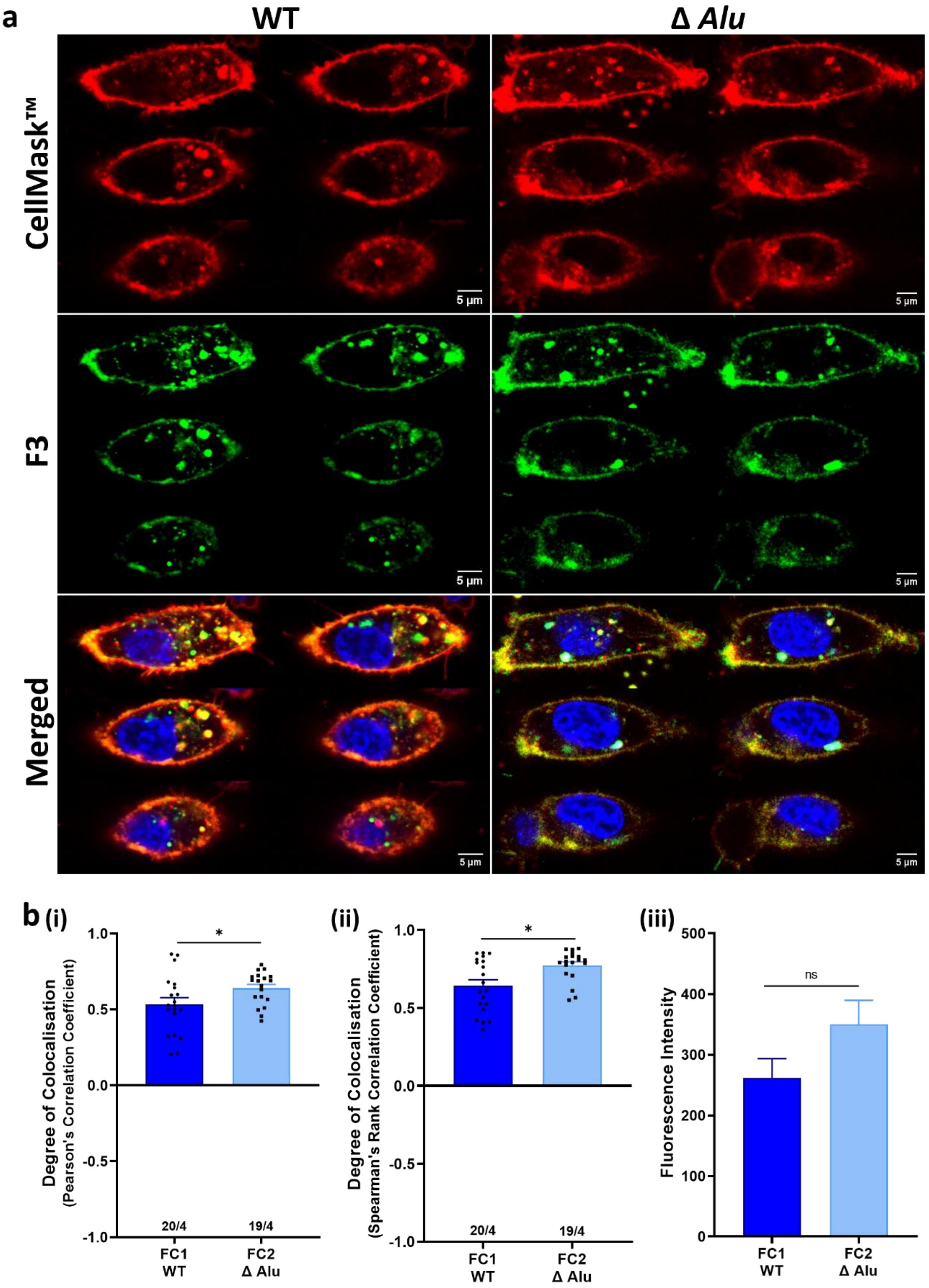

Figure S2. Removal of the *Alu* element in the *F3* 3'-UTR increases localisation of fluorophore to the plasma membrane.

a. Fluorescence confocal microscopy of live MDA-MB-231 cells after transfection of mNG-WT (green, left panels) or mNG-Δ*Alu* (green, right panels), and stained with CellMask™ Orange visualising plasma membrane (red), and Hoechst 33342 (blue). The top panel shows plasma membrane, the middle column shows the fluorescent F3 constructs, and the bottom panel shows a merged image. Any colocalisation of F3 with plasma membrane would appear yellow. Removal of the *Alu* element in F3s 3'-UTR increases the localisation of F3 to the plasma membrane. Images were acquired as a Z-stack using Plan-Apochromat 63x/1.40 Oil DIC M27 lens on Zeiss LSM-780 inverted confocal

microscope. Shown are 6 representative slices of the Z-stack. Representative of multiple images. Scale bars, 5 µm.

b. Pearsons (i) and Spearman's rank (ii) for the correlation of colocalisation of the plasma membrane with mNeonGreen protein under the influence of F3's WT and Δ*Alu* 3'-UTR are shown, along with the mean intensity of the fluorescent constructs (iii). The number of cells analysed/the number of independent experiments is indicated on the x axis. Data are expressed as mean ± SEM and analysed using two-tailed Student's t-test. *$p<0.05$.

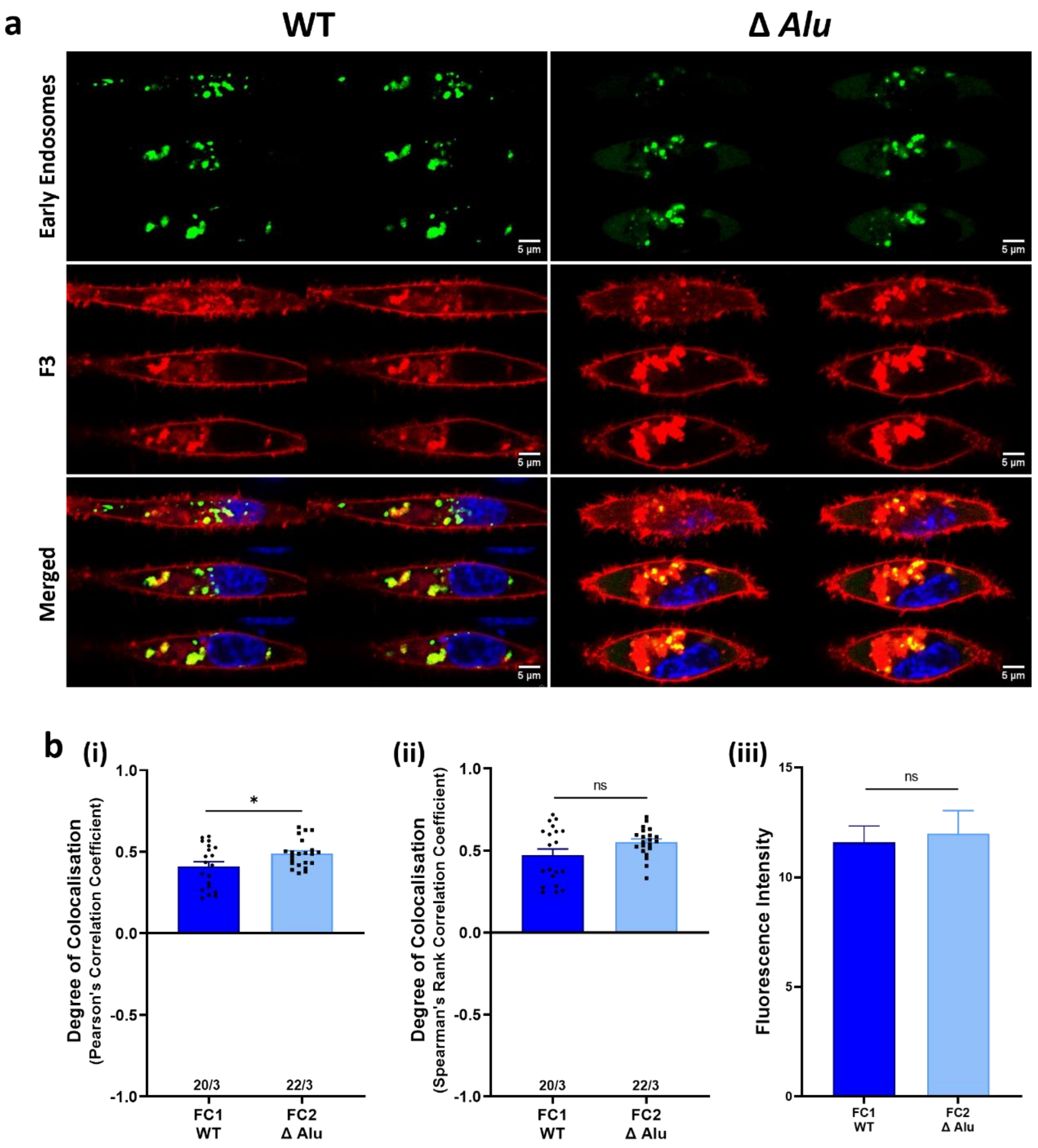

Figure S3. Removal of the *Alu* element in the *F3* 3'-UTR increases fluorophore localisation to early endosomes.

a. Fluorescence confocal microscopy of live MDA-MB-231 cells after transfection of mCh-WT (red, left panels) or mCh-ΔAlu (red, right panels), with GFP-EEA1 visualising early endosomes (green), and stained with Hoechst 33342 (blue). The top panel shows the early endosomes, the middle column shows the fluorescent F3 constructs, and the bottom panel shows a merged image. Any colocalisation of F3 with early endosomes would appear yellow. The ΔAlu localised with early endosomes, but this is lost with the presence of the Alu element. Images were acquired as a Z-stack using Plan-Apochromat 63x/1.40 Oil DIC M27 lens on Zeiss LSM-780 inverted confocal microscope. Shown are 6 representative slices of the Z-stack. Representative of multiple images. Scale bars, 5 µm.
b. Pearsons (i) and Spearman's rank (ii) for the correlation of colocalisation of early endosomes with mCherry protein under the influence of F3's WT and ΔAlu 3'-UTR are shown, along with the mean intensity of fluorescent constructs (iii). The number of cells analysed/the number of independent experiments is indicated on the x axis. Data are expressed as mean ± SEM and analysed using two-tailed Student's t-test. *$p<0.05$.

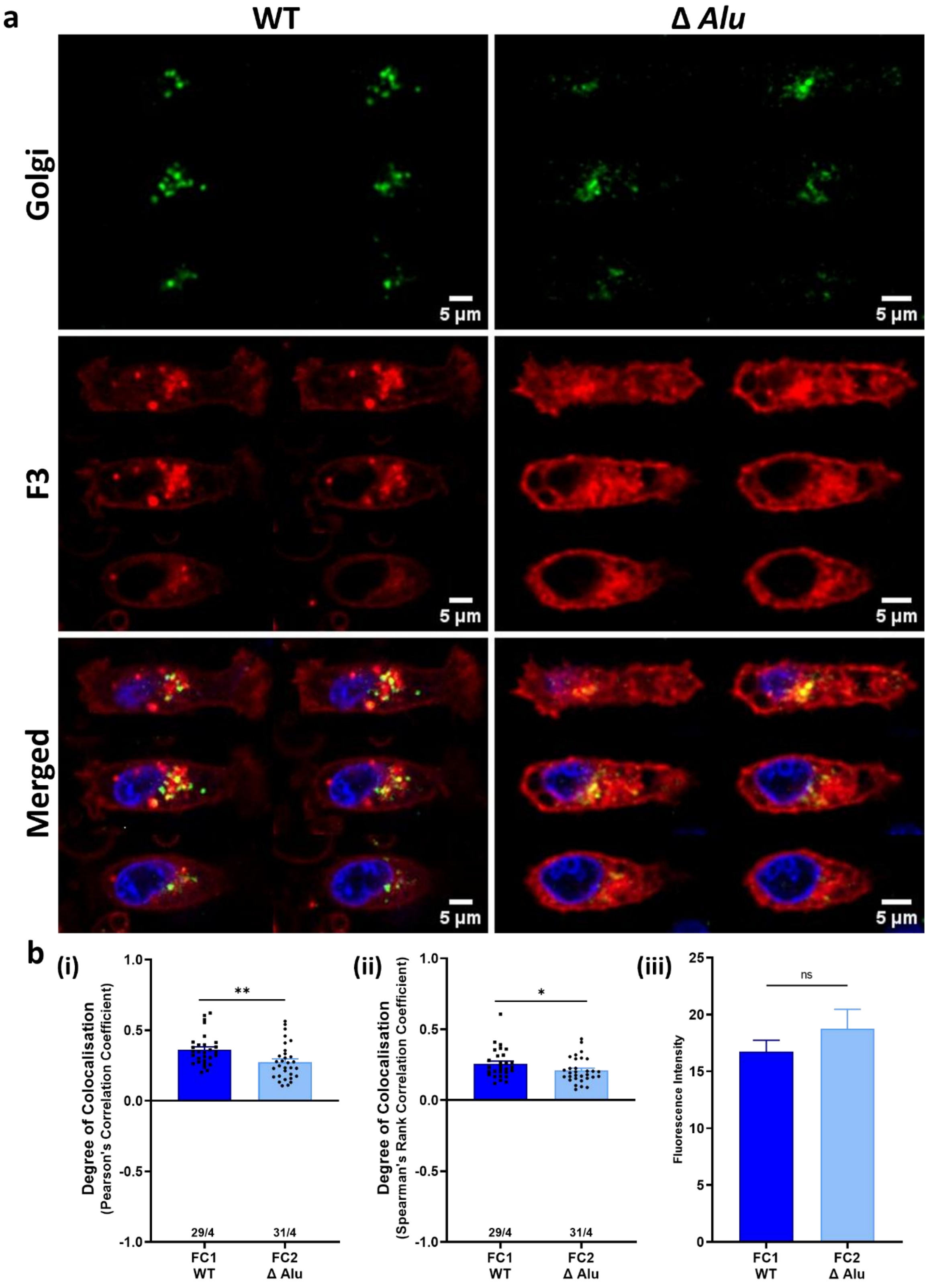

Figure S4. The presence of the *Alu* element in the *F3* 3'-UTR increases the localisation of fluorophore to the Golgi.

a. Fluorescence confocal microscopy of live MDA-MB-231 cells after transfection of mCh-WT (red, left panels) or mCh-Δ*Alu* (red, right panels), with PA-GFP Golgi visualising the Golgi (green) and stained with Hoechst 33342 (blue). The top panel shows the Golgi, the middle column shows the fluorescent F3 constructs, and the bottom panel shows a merged image. Any colocalisation of F3 with the Golgi would appear yellow. The addition of the *Alu* element in F3s 3'-UTR increases the localisation to the Golgi. Images were acquired as a Z-stack using Plan-Apochromat 63x/1.40 Oil DIC M27 lens on Zeiss

LSM-780 inverted confocal microscope. Shown are 6 representative slices of the Z-stack. Representative of multiple images. Scale bars, 5 µm.

b. Pearsons (i) and Spearman's rank (ii) for the correlation of colocalisation of the Golgi with mCherry protein under the influence of F3's WT and Δ*Alu* 3'-UTR are shown, along with the mean intensity of the fluorescent constructs (iii). The number of cells analysed/the number of independent experiments is indicated on the x axis. Data are expressed as mean ± SEM and analysed using two-tailed Student's t-test. *$p<0.05$ **$p<0.01$.

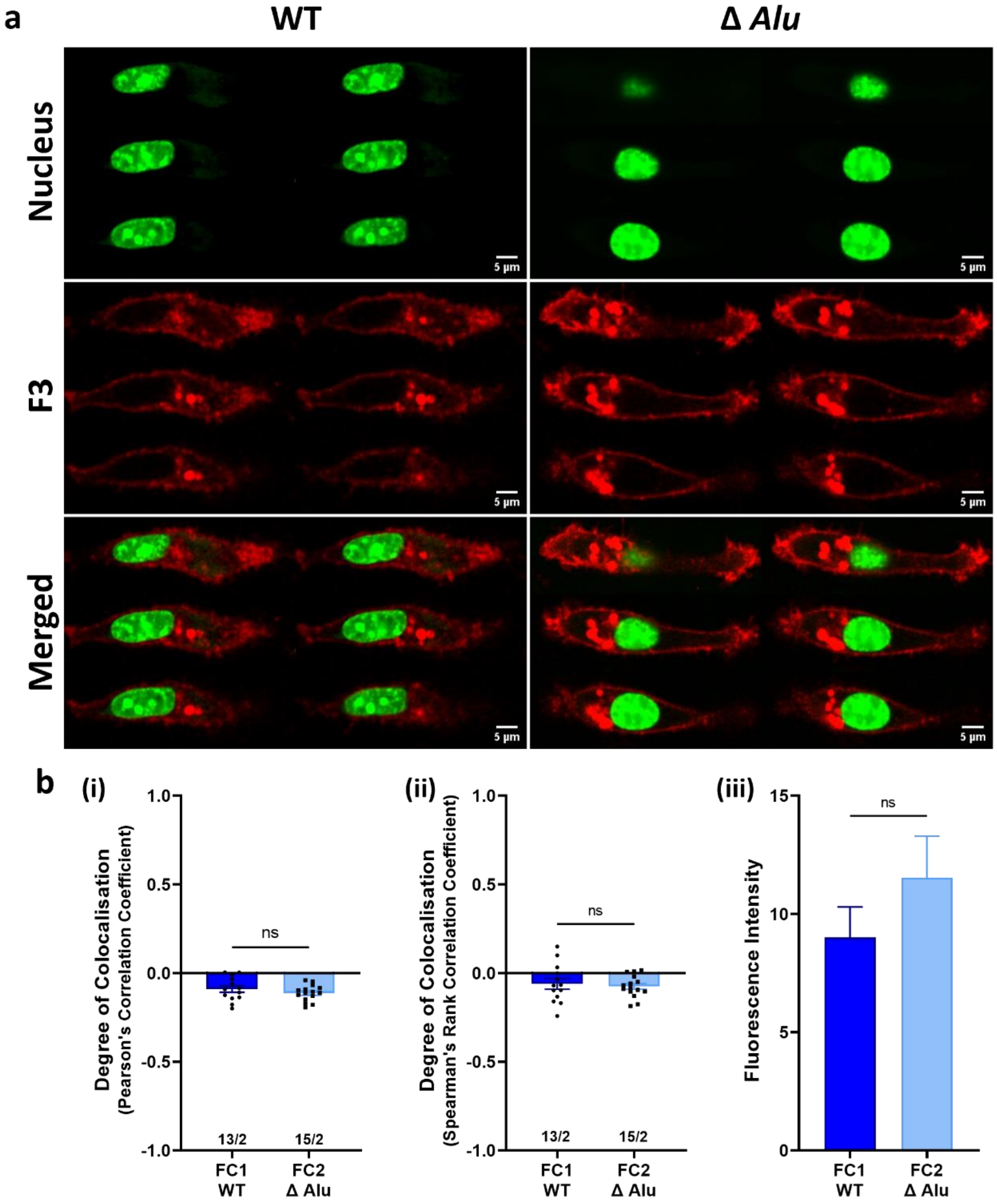

Figure S5. The fluorophores do not localise to the nucleus, regardless of the *Alu* element insertion.

a. Fluorescence confocal microscopy of live MDA-MB-231 cells after transfection of mCh-WT (red, left panels) or mCh-Δ*Alu* (red, right panels), with mEmerald-nucleus-7 (green). The top panel shows the nucleus, the middle column shows the mCh-WT / mCh-Δ*Alu* constructs, and the bottom panel shows a merged image. Images were acquired as a Z-stack using Plan-Apochromat 63x/1.40 Oil DIC M27 lens on Zeiss LSM-780 inverted confocal microscope. Shown are 6 representative slices of the Z-stack. Representative of multiple images. Scale bars, 5 µm.
b. Pearsons (i) and Spearman's rank (ii) for the correlation of colocalisation of the nucleus with mCherry protein under the influence of F3's WT and Δ*Alu* 3'-UTR are shown, along with the mean intensity of the fluorescent constructs (iii). The number of cells analysed/the number of independent experiments is indicated below the bars. Data are expressed as mean ± SEM and analysed using two-tailed Student's t-test.

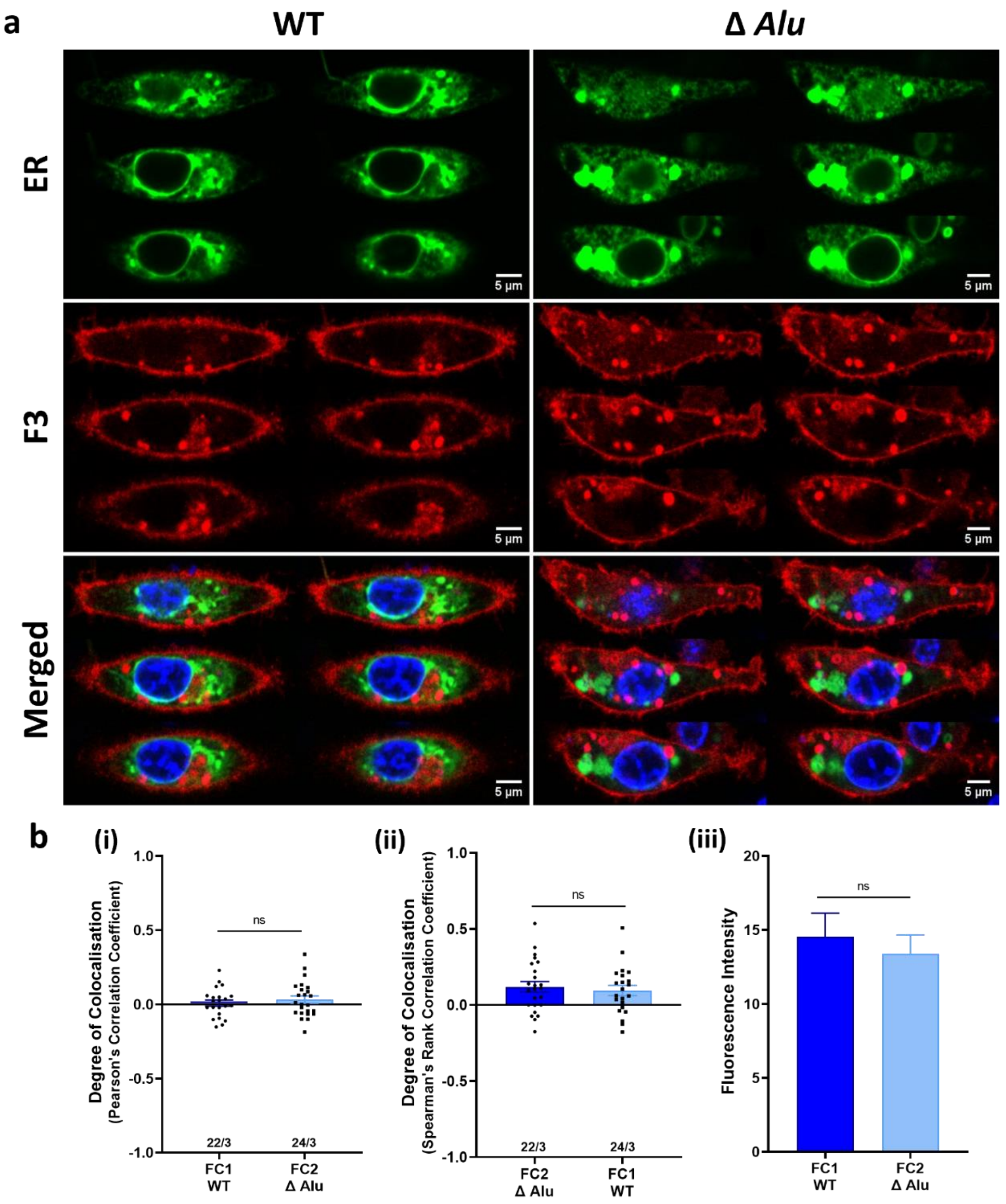

Figure

Figure S6. The fluorophores do not localise to the endoplasmic reticulum, regardless of the *Alu* element insertion.

a. Fluorescence confocal microscopy of live MDA-MB-231 cells after transfection of mCh-WT (red, left panels) or mCh-Δ*Alu* (red, right panels), with GFP-SEC61B visualising the ER (green) and stained with Hoechst 33342 (blue). The top panel shows the ER, the middle column shows the fluorescent F3 constructs, and the bottom panel shows a merged image. Images were acquired as a Z-stack using Plan-Apochromat 63x/1.40 Oil DIC M27 lens on Zeiss LSM-780 inverted confocal microscope. Shown are 6 representative slices of the Z-stack. Representative of multiple images. Scale bars, 5 µm.
b. Pearsons (i) and Spearman's rank (ii) for the correlation of colocalisation of the ER with mCherry protein under the influence of F3's WT and Δ*Alu* 3'-UTR are shown, along with the mean intensity of fluorescent constructs (iii). The number of cells analysed/the number of independent experiments is indicated below the bars. Data are expressed as mean ± SEM and analysed using two-tailed Student's t-test.
